# Designing and Implementation of a Digitalized Intersectoral Discharge Management System and Its Effect on Readmissions: Mixed Methods Approach

**DOI:** 10.2196/47133

**Published:** 2024-03-26

**Authors:** Christoph Strumann, Lisa Pfau, Laila Wahle, Raphael Schreiber, Jost Steinhäuser

**Affiliations:** 1 Institute of Family Medicine University Medical Centre Schleswig-Holstein, Campus Lübeck Lübeck Germany; 2 Lacanja GmbH Health Innovation Port Hamburg Germany

**Keywords:** digitalization, intersectoral, discharge management, readmission, mixed methods design

## Abstract

**Background:**

Digital transformation offers new opportunities to improve the exchange of information between different health care providers, including inpatient, outpatient and care facilities. As information is especially at risk of being lost when a patient is discharged from a hospital, digital transformation offers great opportunities to improve intersectoral discharge management. However, most strategies for improvement have focused on structures within the hospital.

**Objective:**

This study aims to evaluate the implementation of a digitalized discharge management system, the project “Optimizing instersectoral discharge management” (SEKMA, derived from the German Sektorübergreifende Optimierung des Entlassmanagements), and its impact on the readmission rate.

**Methods:**

A mixed methods design was used to evaluate the implementation of a digitalized discharge management system and its impact on the readmission rate. After the implementation, the congruence between the planned (logic model) and the actual intervention was evaluated using a fidelity analysis. Finally, bivariate and multivariate analyses were used to evaluate the effectiveness of the implementation on the readmission rate. For this purpose, a difference-in-difference approach was adopted based on routine data of hospital admissions between April 2019 and August 2019 and between April 2022 and August 2022. The department of vascular surgery served as the intervention group, in which the optimized discharge management was implemented in April 2022. The departments of internal medicine and cardiology formed the control group.

**Results:**

Overall, 26 interviews were conducted, and we explored 21 determinants, which can be categorized into 3 groups: “optimization potential,” “barriers,” and “enablers.” On the basis of these results, 19 strategies were developed to address the determinants, including a lack of networking among health care providers, digital information transmission, and user-unfriendliness. On the basis of these strategies, which were prioritized by 11 hospital physicians, a logic model was formulated. Of the 19 strategies, 7 (37%; eg, electronic discharge letter, providing mobile devices to the hospital’s social service, and generating individual medication plans in the format of the national medication plan) have been implemented in SEKMA. A survey on the fidelity of the application of the implemented strategies showed that 3 of these strategies were not yet widely applied. No significant effect of SEKMA on readmissions was observed in the routine data of 14,854 hospital admissions (*P*=.20).

**Conclusions:**

This study demonstrates the potential of optimizing intersectoral collaboration for patient care. Although a significant effect of SEKMA on readmissions has not yet been observed, creating a digital ecosystem that connects different health care providers seems to be a promising approach to ensure secure and fast networking of the sectors. The described intersectoral optimization of discharge management provides a structured template for the implementation of a similar local digital care networking infrastructure in other care regions in Germany and other countries with a similarly fragmented health care system.

## Introduction

### Background

Digital patient process systems offer several advantages over analog systems. On the one hand, this can lead to more systematic, targeted use of resources, and on the other hand, easier communication and transmission of data can enable better coordination of the various cooperating partners [1]. Patient records are becoming increasingly digitalized, with some countries being prototypes in this area, such as Latvia, Denmark, and Spain [2].

In Germany, there have been several governmental attempts to shape different elements of health care digitalization. A recent example is the Hospital Future Act (Krankenhauszukunftsgesetz) from 2020. It was designed to support digitalization in hospitals by promoting the technical equipment of hospitals through state-funded investments. The investments are expected to improve process organization, documentation, and communication (internal, sectoral, and intersectoral) [3]. The results suggest that the Hospital Future Act, together with the COVID-19 pandemic, led to an increase in the digital maturity of hospitals and, thus, reduced the digitalization backlog [4]. Another approach to promote health care digitalization is the introduction of an electronic health record (EHR) within a secure telematics infrastructure. The EHR should not only simplify rapid communication within and across different health care institutions but also enable further eHealth applications, for example, electronic prescriptions [5]. However, the introduction of EHRs as well as other reforms promoting health care digitalization have been accompanied with strong resistance underpinned by arguments of data protection and security as well as by technical problems. Especially in the outpatient sector, the latter has resulted in a perceived disproportionate administrative effort without adequate financial compensation for the care providers such as private practices [6]. As a result, Germany lags behind other industrialized countries in the digitalization of the health care system [7,8].

An EHR could make treatment pathways more transparent and improve communication between different health care providers, including inpatient, outpatient and care facilities [9]. The exchange of information is particularly susceptible if a patient is discharged from hospital. With regard to the strongly pronounced sectoral separation in Germany [10,11], information loss is particularly high between inpatient and outpatient care. Moreover, owing to the accelerated tendency toward shortening the length of stay of patients in the inpatient sector as a result of the introduction of the diagnosis-related group–based reimbursement system [12], hospitals no longer provide care and treatment until full recovery [13]. Instead, parts of the treatment and recovery process are moved to the posthospital setting [14]. Similar developments have been observed after introducing the diagnosis-related group–based reimbursement system in other countries, for example, the United States [15-17]. Shortened length of stay and ineffectively designed transitions are associated with adverse events, higher risks of readmission, and higher costs [18-21]. Up to 1 (18%) in 5 patients are readmitted to the hospital within 30 days of discharge [22,23]. Individualized discharge management can reduce the number of readmissions of older patients with a health problem [24], leading to potential cost savings for the health care system [25]. To date, many strategies to improve discharge management have focused on structures within the hospital. However, to ensure a holistic and continuous treatment, the cooperation between different health care providers from the inpatient and outpatient sectors as well as care facilities should also be considered.

As there is still no EHR accessible to all caregivers in Germany, experience with digitalized health information systems has been gathered only in model projects, which are intended to provide insights into possible barriers and enablers for a successful implementation [5,26-30].

### Objectives

This study aims to explore the determinants of a digitalized discharge management system, to implement such a system within 1 area, and to evaluate its impact on the readmission rate.

## Methods

The evaluation was done within the project “Optimizing intersectoral discharge management” (SEKMA, derived from the German Sektorübergreifende Optimierung des Entlassmanagements).

### Study Design

A mixed methods design was chosen to evaluate SEKMA. Owing to the complexity of the intervention, the evaluation was based on the framework of developing tailored interventions [31]. This approach allows a detailed description and analysis of the components of the intervention that contributed to its effectiveness or ineffectiveness. For this purpose, this framework distinguishes between a development and an application phase. In the first step, barriers and enabling factors for a successful implementation of a digitalized discharge management system such as SEKMA were explored using qualitative research methods, that is, interviews. Second, strategies were developed for addressing these determinants. Third, these strategies were prioritized using a (quantitative) questionnaire, and a logic model was formulated to describe the logical linkages among the resources and activities needed to achieve the results. After the implementation (application phase), the congruence between the planned intervention (logic model) and the implemented intervention was evaluated. In this step, the fidelity of the use of the different strategies in the routine was examined [32]. Finally, the effectiveness of the implementation on the readmission rate (outcome) was evaluated based on routine data of hospital admissions.

### Setting

The digitalized discharge management system was implemented at a medium-sized hospital (approximately 350 beds) in the northern German federal state Schleswig-Holstein in the Metropolitan area of Hamburg, the second-largest city in Germany. Before the intervention, the internal and external exchange of information was typically performed by phone, fax, and email. As the network between the various caregivers was rather weak, communication occurred only on request, tying up resources and causing delays in the transfer of information.

SEKMA aimed to develop and implement a digitalized, intersectoral discharge management system that considers the patient’s entire treatment pathway, from hospital admission to possible admission to a care facility, and the follow-up treatment by general practitioners (GPs). All information relevant to ongoing (postinpatient) treatment and care should be available quickly and easily to all care providers involved. This includes providers from the inpatient and outpatient sectors as well as care facilities. For this purpose, an ecosystem of hospital and postinpatient care facilities has been implemented within a digital infrastructure based on a standardized and harmonized IT system for data exchange [33]. The workflow of the digitalized, intersectoral discharge management can be described as follows:

1. The hospital coordinates and organizes follow-up care in a timely manner based on the patient’s agreement with the hospital’s discharge management.
2. A discharge plan for medication, follow-up care, and rehabilitation is created and all professionals in the hospital are involved. This includes admission staff, medical service, nursing service, social service, and the patient information system.
3. In cooperation with the nursing staff and social service, the patient is informed and advised about care options and structures that correspond to their illness. The contents are prepared digitally.
4. The patient is discharged from the hospital and transitions to outpatient, rehabilitative, or nursing care. All documents necessary for discharge and further treatment are available digitally and can be transmitted directly to the relevant sectors.
5. If a patient contacts a primary care physician for outpatient follow-up treatment, the patient’s digital discharge documentation is already in the system of the private practice.
6. In case of a query or deterioration of health status, the primary care physician can contact the hospital and previously treating physicians directly.
7. If there is a readmission, the hospital can digitally access documentation on posttreatment care and procedures, as well as the medical history, at any time and continue treatment directly. The same applies to nursing and rehabilitation facilities.

The information transfer across the distinct health care provider is organized via KIM (Kommunikation im Medizinwesen) embedded in the telematics infrastructure. All organizations involved in the project have a KIM connection. Using KIM, participants can transmit documents in a secure and encrypted manner [34]. Overall, all communication processes have been digitalized compared with before the intervention. Since April 2022, optimized discharge management has been implemented in the department of vascular surgery.

### Individual Interviews

Enabling factors and barriers leading toward successful digital discharge management were identified through individual interviews with physicians, medical assistants, social workers and nurses at the hospital, GPs, and staff from nursing homes and care services. This was performed using the COREQ (Consolidated Criteria for Reporting Qualitative Research) guidelines for qualitative studies (Multimedia Appendix 1 provides details of the COREQ guidelines [35]). Originally, a combination of interviews and focus groups was planned. Owing to the COVID-19 pandemic, focus groups had to be abandoned.

The hospital, along with collaborating partners such as physician networks and nursing homes, conducted participant recruitment for interviews through face-to-face interactions, telephone calls, and emails. Previously developed partially standardized interview guidelines were used and pilot tested (Multimedia Appendix 2). The interviews were conducted by telephone by a medical student (LP) between April 30, 2020, and October 9, 2020, at the workplace of the interviewees. A theoretical saturation effect in the statements made during the interviews resulted in the final number of interviewees.

The individual interviews were conducted in a protected setting and subsequently pseudonymized, thus providing the opportunity to explore the personal opinions of the interviewees beyond any possible social group pressures. The interviews were recorded using a digital dictaphone and were transcribed orthographically. The material was subsequently analyzed using structured content analysis according to Mayring [36]. The development of the categories was initially based on the questions (deductive) listed in the partially standardized interview guideline (Multimedia Appendix 2). In addition, categories were extracted from the text (inductive). Five persons were involved in the development of the category scheme (LP: medical student [female researcher], JS: GP and experienced health service researcher including qualitative research [male researcher], CS: health economist with some experience in qualitative research [male researcher], a legal project advisor [female researcher], and a physiotherapist [female researcher]; all of them except LP were employed at the Institute of Family Medicine at the University of Lübeck at the time of the analysis). After individual coding, a coding scheme was discussed in a consensus meeting. The final coding scheme was applied to the interview material.

### Development and Evaluation of Strategies

On the basis of the described processes for treating the patients, the optimization potential, and the determinants from the evaluated individual interviews as well as the workshop with clinicians and physicians in private practice, strategies for the implementation of optimized discharge management were developed. These strategies were developed in such a way that they addressed the determinants identified and were, thus, conducive to a successful implementation.

During a project meeting on February 3, 2022, employees of the Institute of Family Medicine at the University of Lübeck and the chief and senior physicians of the involved hospital discussed these results. Subsequently, the hospital’s chief or senior physicians were invited to evaluate each identified strategy according to its relevance and feasibility using a 6-point Likert scale (very high, rather high, high, rather low, low, and very low) to avoid the central tendency bias.

The resulting list of the ranked strategies formed the logic model. This model was finally compared with the list of strategies implemented in the project.

### Routine Data Analysis

The focus of the evaluation of the optimized discharge management was the reduction of (unnecessary) readmissions. With the help of the evaluation of the routine admission data of the involved hospital, the effect of optimized discharge management on rehospitalization was analyzed.

#### Routine Data and Study Design

The hospital extracted routine data from its internal patient information system. The extracted data were provided by the hospital in an anonymized form. For each inpatient case, the data consisted of information on the date of admission and discharge, the reason for admission and discharge, diagnoses and conducted medical procedures, demographic information of the patients, and the department or departments where the patients had been treated.

Within the framework of a longitudinal study design, a pre- and postcomparison was performed. The intervention group was the department of vascular surgery, in which the optimized discharge management was implemented since April 2022. A case was assigned to the intervention group if the patient was admitted to or discharged from the department of vascular surgery. The outbreak of COVID-19 during the sample period might have affected the readmissions of the entire hospital. To minimize the risk of bias owing to the pandemic on the intervention effect, in addition to the pre-post comparison of the department of vascular surgery, a control group comparison was applied to enrich the empirical strategy. To ensure that the patients in the intervention group were as similar as possible to those in the control group, the departments of internal medicine (medical clinic) and cardiology formed the control group.

#### Statistical Analysis

The effect of the implementation was estimated using the difference-in-difference (DiD) approach. The sample covers the period from 2019 to August 2022. To counteract the possible COVID-19 pandemic bias, patients admitted between January 2020 and March 2022 were not considered in the analysis. To avoid any seasonal influences on the results, we restricted the preintervention period such that it covered exactly the period after the implementation, that is, from April to August. Therefore, the baseline period (T_0_) consisted of April 1, 2019, to August 31, 2019, whereas the intervention period (T_1_) started from April 1, 2022.

In addition to the bivariate analysis, a multivariate logistic regression model was applied. By including control variables, differences between patients from the intervention and control group were minimized. In the first step, risk factors for rehospitalization were determined by estimating separate bivariate logistic regression models. The identified risk factors served as control variables in the multivariate DiD regression analysis. A *P* value <.05 was considered statistically significant. Statistical analyses were performed with Stata (version 15; StataCorp LLC).

### Ethical Considerations

The study was approved by the ethics committee of the University of Lübeck before recruitment commenced on December 11, 2019 (approval number 19-387). This study was conducted in accordance with the Declaration of Helsinki.

All participants provided verbal and written informed consent for their participation in the interviews and surveys. The participants were informed that they could withdraw their consent at any time. No identifiable information was recorded to ensure the confidentiality of the participants. No compensation was paid for participation.

For the analysis of routine hospital data, only anonymized data were transferred to the evaluating institution. Owing to the anonymization of the data, no additional informed consent was required to perform the routine data analysis in accordance with German law, ethical standards, and the Declaration of Helsinki. No data requiring informed consent will be presented in the routine data analysis. The ethics committee of the University of Lübeck waived the requirement for informed consent owing to the retrospective nature of this study.

## Results

### Interviews

#### Sample

A total of 26 interviews were conducted. These consisted of 14 employees of the hospital (3 doctors, 4 nurses, 4 social workers, and 3 administrative staff), 9 employees from nursing homes or mobile nursing services, and 3 GPs. The average age of the participants was 42.4 (SD 8.9; range 25-65) years, and the proportion of female participants was 54% (14/26). The average interview duration was 33 minutes and 11 seconds. An overview of the characteristics of the interview participants is provided in Table S1 in Multimedia Appendix 3.

#### Categories

A total of 21 determinants were explored with various subcategories for the introduction of successful digitalized discharge management. These could be divided into 3 categories: “optimization potential,” “barriers,” and “enablers.” The aspects mentioned for optimizing the discharge process covered all areas from admission to follow-up and included inter- and intrasectoral transmission of information (Textbox 1).

**Potential for optimization of discharge management.**

**Category and subcategories**

- Transmission of information
  - Preliminary discharge letter before discharge
  - Final discharge letter at the time of discharge
  - Digital transmission (mail, chat, and video call)
  - Platform for information exchange
  - Standardized information
  - Increased readiness to communicate
- Admission
  - Information exchange at admission
  - Consent to discharge management
- Preparation for discharge
  - Awareness of the existence of discharge management in the hospital
  - Timely completion of the discharge process
  - Continuous preparation for (unplanned) discharge
  - Improvement of patient communication
  - Faster approvals by health insurances
- Discharge
  - Discharge in the morning of the working day
  - Material transfer, issuing of prescriptions and incapacity certificate
  - Nursing services accompany discharge from hospital
  - Increase in the availability of patient transport
- Aftercare
  - Visits to general practitioner after discharge
  - More aftercare places
- Process standardization (standard operating procedure)
  - Training on discharge
  - Digital checklist
  - Standardized processes
  - Clarified responsibilities
- Intersectoral optimization
  - Knowledge of the performance and processes at other facilities
- Structural
  - Evaluation of criticism or review
  - Supervision
  - Ethics committee

In the German health care system, the discharge letter is at the center of information transmission between the inpatient and outpatient sectors. Participants saw a need for improvement in the early, or at least timely, delivery of this letter. In the best case, information would already be transmitted during the hospital stay to the follow-up service providers such as private practices or care facilities:

To have all the information and data, everything before the patient arrives here. That would be the absolute dream. [...] You can just admit the person better[...] if you just have preliminary information.

P03

Digital transmission of data was also perceived as beneficial; the participants could imagine using conventional media such as email or video calls as well as via a platform provided specifically for this purpose:

If you could even find some other common platform where information can be exchanged.

P01

Furthermore, the potential for optimization was seen in the standardization of the information. The information to be communicated should be transmitted through a central entity, and at the same time, selected contacts who can be reached on demand and who can provide information about the patients would be beneficial:

Yes, standard, standard, standard. So, that you try to agree on what information I need and then it has to appear—in a structured form, so in principle already like my patient information.

P10

Some participants also noted that, in principle, a greater willingness to communicate between the individual players would improve the transmission of information.

Participants noted that for a seamless discharge, information about the patient should already be available at the time of admission to the hospital:

Discharge or discharge planning and a good discharge process starts at admission. [...] The important thing is not to think about discharge on the day of discharge, but already on admission.

P25

Improved patient communication was also considered important by interviewees:

And that is certainly a wish that I would have that the patients in the hospital are also informed about what they actually have, what has happened and what the next steps are.

P01

An optimal discharge should ideally take place in the morning on a working day, and the handing over of medication and required materials should be regulated. This is considered to be the case by nursing homes and outpatient care services as well as by hospital staff:

From 9 or 10 a.m onwards, the number of patients in the emergency room increases and drops again from 8 p.m onwards. And during this peak time, there are few beds available in the hospital. Afterwards, however, when we are closed, the hospital finally loses cases and at night we have more free capacity again. And that is a mismatch between demand and capacity which can be improved.

P20

Textbox 2 shows the barriers and enabling factors for intersectoral collaboration in the context of optimizing discharge management. In addition to the technical aspects and subjective reasons, there were concerns about data protection and fear that a change in the discharge process would require more time:

Time pressure is always an issue, both in the hospital and in outpatient care. We just often don’t have the time for some processes that we would all consider useful.

P01

**Determinants for intersectoral cooperation.**

**Barriers**

- Privacy
  - Data transmission security
  - Legal uncertainties
- Fear of contact
  - Leaving known structures and processes
  - Lack of electronic data processing experience
- Lack of time
  - Higher time consumption
  - Lack of personnel
- Subjective reasons
  - Limitation of one’s own competence
  - Unclear communication processes
  - No perceived benefit
  - Low appreciation for discharge management
  - No priority of discharge management
  - No consequences for noncompliance
- Technical aspects
  - User-unfriendly system
  - Electronic data processing errors
  - Interface problems
  - Outdated technical equipment
- Change processes
  - Lack of education or communication
  - Lack of networking among health care providers

**Enablers**

- Structures
  - Clear responsibilities, instructions, contact persons, or responsibilities
  - Surveillance
  - Introduction or training of new processes
  - No overload and enough time
  - Regular exchange for networking
- Recognizable advantage
  - Time saving
  - Workload reduction
  - Improved exchange of information
  - Feedback loops
- Introduction of change management
  - Priority in the management
  - Communicating the advantages
  - Involvement of employees

In contrast, a possible reduction in workload owing to digitalized processes was seen as conducive:

Digitalization must not be an end in itself, in my opinion, but it must really mean an advantage for the processes, increase safety, increase communication, but it must not be a question of just because it is digital, that it is better in every case and is then associated with the fact that medical or nursing working time is lost or additionally created.

P23

For the changeover to be successful, the communication of the advantages associated with optimized discharge management was emphasized above all as part of change management.

### Strategies

On the basis of the surveyed processes, the optimization potential, and the determinants from the evaluated individual interviews as well as the workshop with clinicians and physicians in private practice, 19 strategies for the implementation of optimized discharge management were developed. To rank these strategies, chief physicians of the hospital were invited to rate their relevance and feasibility.

A total of 11 physicians participated in the survey to evaluate the strategies (Table 1). The strategies of always sending the discharge letter to the GP, equipping the hospital’s social service with mobile devices (eg, laptops and tablets), generating individual medication plans in the format of the national medication plan, and exclusively using the federal medication plan received the highest ratings. In contrast, the introduction of a chat function used exclusively by physicians for direct exchange between hospital and office-based physicians received the lowest rating.

**Table 1:** Evaluation of the strategies (N=11).

Strategy	Sum^a^, mean (SD)	Relevance^a^, mean (SD)	Feasability^a^, mean (SD)	Implemented intervention
Always send the discharge letter to the GP^b^	11.1 (1.4)	5.7 (0.7)	5.4 (0.8)	Yes
Providing mobile devices to the hospital’s social services department (eg, laptop and tablet)	10.9 (1.5)	5.6 (0.7)	5.3 (0.9)	Yes
Generating individual medication plans in the format of the national medication plan	10.8 (1.3)	5.6 (0.7)	5.2 (0.8)	Yes
Exclusive use of the national medication plan	10.6 (1.8)	5.5 (0.7)	5.1 (1.3)	No^c^
Development of digital checklists for discharge processes to be integrated into clinic software	9.9 (1.6)	5.3 (0.9)	4.6 (1.3)	No^c^
Standardized consent process for discharge management at the time of admission	9.8 (3.1)	5.0 (1.6)	4.8 (1.5)	Yes
Sending or receiving an electronic discharge letter (eg, compatible with the electronic patient record)	9.7 (2.0)	5.2 (1.1)	4.5 (1.1)	No^c^
Always send the discharge letter to the patient as a message	9.6 (1.8)	4.3 (1.3)	5.3 (1.0)	No
Organization of necessary (postdischarge) follow-up treatments (eg, by a medical assistant employed at the hospital)	9.5 (2.6)	5.1 (1.4)	4.4 (1.4)	No
Preventing multiple information collection through automated exchange within the different clinic software	9.2 (1.8)	5.7 (0.9)	3.5 (1.5)	No^c^
Conduct training for all people involved in the process to raise awareness of the relevance of discharge management	9.0 (2.1)	4.8 (1.3)	4.2 (1.2)	No^c^
Upon discharge, information is sent to the GP about which medications on the medication plan have changed, if any	9.0 (1.7)	5.2 (0.6)	3.8 (1.2)	No
Ensuring that patients have had at least 1 complete physical examination before discharge	9.0 (2.6)	5.0 (1.1)	4.0 (1.7)	No
Medication plan is available to the GP on the morning of the day of discharge (eg, sent electronically in advance)	8.9 (2.0)	5.0 (0.9)	3.9 (1.3)	No^c^
Hotline used exclusively by physicians for direct exchange between hospital and GPs	8.8 (2.3)	5.2 (0.9)	3.6 (1.6)	Yes
Reasons for medication changes in the medication plan, if \applicable, are communicated to the GP at discharge	8.5 (2.2)	4.9 (1.0)	3.6 (1.3)	No
Continuous digital maintenance of a potential discharge letter (eg, in preparation for an unplanned discharge)	8.1 (2.7)	4.3 (1.3)	3.8 (1.5)	Yes
Establishment of a quality circle for discharge management	7.9 (2.6)	4.0 (1.5)	3.9 (1.4)	No
Introduction of a chat function used exclusively by physicians for direct exchange between the hospital and GPs	7.0 (2.6)	3.9 (1.5)	3.1 (1.2)	No^c^

^a^Mean over participants (6=very high, 5=rather high, 4=high, 3=rather low, 2=low, and 1=very low).

^b^GP: general practitioner.

^c^Not part of the intervention but planned for the future by the hospital.

On the basis of these ratings, the hospital staff discussed which of these strategies were already being implemented or planned for implementation in the near future. Of the 19 strategies, 6 (31%) were assessed as already implemented, 7 (37%) were assessed as planned, and 6 (31%) were assessed as not feasible to implement in the project. The 7 strategies rated highest in the development of the logic model (planned implementation) have been implemented or will be implemented in the near future as part of SEKMA.

To summarize, by April 2022, at the department of vascular surgery, (1) discharge letters were continuously updated digitally, (2) they were always sent, (3) they were sent electronically to the GP (via the infrastructure of KIM), (4) the hospital social service was equipped with mobile devices, (5) individual medication plans were in the format of the national medication plan, (6) the discharge management consent process at admission was standardized, and (7) a hotline for direct communication between hospital physicians and primary care physicians was implemented. The information transfer via the discharge letter was oriented by the standard of medical information objects (MIOs) eArztbrief. The development of this standard was initiated in 2022 by the National Association of Statutory Health Insurance Physicians and the German Hospital Association. It defines a standard for the electronic hospital discharge letter within the EHR ensuring the transition of relevant information from inpatient to subsequent care in a structured and secure manner [37]. The MIO eArztbrief was not yet ready during the project; however, the current status of the MIO was incorporated into the letter as much as possible.

### Fidelity Analysis

After the implementation of the optimized discharge management into the routine in the department of vascular surgery as well as at the external partners in April 2022, the stakeholders participating in the project were asked in a fidelity analysis in September 2022 to what extent the identified strategies were implemented in practice. The survey showed that many of these strategies were not yet widely applied.

A total of 14 individuals responded to the survey (Table S2 in Multimedia Appendix 2). Of the 14 individuals, 11 (79%) were employed at the hospital and 1 (7%) each at an outpatient nursing service, nursing home, and private practice. Employees from social services and medical assistants did not participate in this survey. Of those surveyed, >30% (4/13) stated that they were satisfied with the implementation of the change in discharge management.

There are differences in the fidelity of use among the strategies implemented (Table S3 in Multimedia Appendix 3). Although sending or receiving an electronic discharge letter was always or sometimes used in their routine by only a quarter of respondents, approximately 85% (11/13) of the respondents indicated that medication plans from the hospital were always in the format of the federal medication plan at discharge.

### Routine Data Analysis

#### Readmissions

In total, 12,407 patients were admitted to the hospital as inpatients during the study period (from April 2019 to August 2019 and from April 2022 to August 2022), corresponding to 14,854 cases treated. The internal medicine department (medical clinic) treated most of the cases (4175/14,854, 28.11%). Cases treated in the interventional group (vascular surgery) accounted for 5.11% (759/14,854) of all inpatient cases. Overall, 8.73% (994/11,386) of the patients were readmitted after 30 days. In terms of treated cases, the readmission rate was 9.07% (1222/13,477). The rates increased to 17.1% (1542/9016) for patients and 18.85% (1975/10,478) for cases when considering a longer time horizon for the readmission (90 days). Readmission rates were generally higher in the intervention group (80/705, 11.3%) at 30 days and 28.8% (161/560 at 90 days) than in the hospital as a whole and the control group. Table S4 in Multimedia Appendix 3 provides the number of admitted patients and cases treated as well as the readmissions after 30, 60, and 90 days for the total hospital cases and the departments involved.

#### Risk Factors

Risk factors for readmission were identified to take the differences between patients from different departments into account for the evaluation of the project’s implementation effect.

Older patients, as well as cases with a length of stay of >6 days, had a significantly higher risk of readmission. Similarly, discharge time influenced the readmission risk: patients discharged during the night (9 PM to 5 AM) had a higher risk of readmission. Similarly, there were significant differences in readmissions between cases with different ICD-10 (*International Statistical Classification of Diseases*, *Tenth Revision*) chapters of principal and secondary diagnoses (Table S5 in Multimedia Appendix 3).

#### Intervention Effect

Table 2 shows the implementation effects on the readmission rate after 30, 60, and 90 days (DiD) of the bivariate analysis. In the intervention group, the 30-day readmission rate increased by 2.33 percentage points from 10.4% (45/431) to 12.8% (35/274) after SEKMA was implemented. For the 60- and 90-day readmission rate, the increase was even higher (60 days: 2.25 and 90 days: 3.94). These increases have been smaller in the control group. Therefore, a reduction effect of the intervention on the readmission rate (ie, a negative DiD estimate) cannot be observed. Concentrating the analysis on patients aged ≥65 years revealed similar results (Table S6 in Multimedia Appendix 3). As a robustness check, the preintervention period was extended to include admissions between 2011 and 2019. These results confirm the previous findings.

**Table 2:** Bivariate analysis of the intervention effect^a^.

			Intervention group: department of vascular surgery		Control group: departments of internal medicine and cardiology		DiD^b^ estimator: Δ (*P* value)
**Readmission 30 days: T_1_ until August 3, 2022**							
	Baseline (T_0_)^c^, n/N (%)	45/431 (10.4)		354/3206 (11.04)		N/A^d^	
	Intervention (T_1_)^e^, n/N (%)	35/274 (12.8)		245/2344 (10.45)		N/A	
	Difference T_0_ and T_1_(*P* value)	2.33 (.35)		–0.59 (.48)		2.92 (.27)	
**Readmission 60 days: T_1_ until July 4, 2022**							
	Baseline (T_0_)^c^, n/N (%)	97/431 (22.5)		549/3206 (17.12)		N/A	
	Intervention (T_1_)^e^, n/N (%)	51/206 (24.8)		302/1761 (17.15)		N/A	
	Difference T_0_ and T_1_ (*P* value)	2.25 (.53)		0.03 (.98)		2.23 (.56)	
**Readmission 90 days: T_1_ until June 4, 2022**							
	Baseline (T_0_)^c^, n/N (%)	120/431 (27.8)		707/3206 (22.05)		N/A	
	Intervention (T_1_)^e^, n/N (%)	41/129 (31.8)		257/1186 (21.67)		N/A	
	Difference T_0_ and T_1_ (*P* value)	3.94 (.40)		–0.38 (.78)		4.32 (.37)	

^a^Only admissions between April 2019 and August 2019 and between April 2022 and August 2022 were considered.

^b^DiD: difference-in-difference, Δ: Difference between the readmission rates of the intervention and the control group at T_0_ and T_1_, respectively.

^c^Intervention period (T_1_): from April 1, 2022.

^d^N/A: not applicable.

^e^Baseline period (T_0_): April 1, 2019, to August 31, 2019.

The results of the multivariate logistic regression model (Table 3) confirm the results of the bivariate analysis that there were higher readmission rates in the intervention group and that there was no significant effect of the optimized discharge management on readmissions in the available data. This result was also confirmed for patients aged >65 years (Table S7 in Multimedia Appendix 3). Furthermore, the insignificance of the effect of the implementation of SEKMA on readmission rates was also confirmed in a pre-post comparison estimated by a multivariate logistic regression based on vascular surgery cases only (Table S8 in Multimedia Appendix 3). Finally, the estimated effects remained very similar if the preintervention period began in 2011 and ended at the end of 2019.

**Table 3:** Multivariate logistic regression^a^.

Variable^b^			Readmissions after				
			30 days: T_1_^c^ until August 3, 2022		60 days: T_1_ until July 4, 2022		90 days: T_1_ until June 4, 2022
**DiD^d^ effect, odds ratio (95% CI)**			1.39 (0.84-2.31)		1.16 (0.76-1.77)		1.27 (0.80-2.02)
	Intervention period, odds ratio (95% CI)	0.96 (0.81-1.15)		1.03 (0.88-1.21)		0.99 (0.84-1.17)	
	Intervention group, odds ratio (95% CI)	0.90 (0.63-1.29)		1.32 (1.01-1.73)		1.29 (1.00-1.66)	
**Social demographics** **, odds ratio (95% CI)**							
	Female	0.93 (0.79-1.09)		0.91 (0.79-1.05)		0.90 (0.78-1.03)	
	Aged >65 years	1.44 (1.17-1.76)		1.41 (1.19-1.67)		1.48 (1.25-1.74)	
**Length of stay (reference: <3 days)** **, odds ratio (95% CI)**							
	3-5	1.51 (1.16-1.95)		1.45 (1.17-1.80)		1.45 (1.18-1.78)	
	6-9	2.12 (1.63-2.76)		2.14 (1.72-2.68)		2.04 (1.65-2.53)	
	>10	2.49 (1.88-3.29)		2.23 (1.76-2.82)		2.25 (1.79-2.83)	
**Transfer within the hospital, odds ratio (** **95% CI** **)**							
	Transfer between departments	0.96 (0.1-1.18)		1.02 (0.85-1.21)		1.06 (0.89-1.25)	
	Intensive care	0.50 (0.31-0.82)		0.69 (0.47-1.00)		0.75 (0.53-1.08)	
**Discharge time (reference: 6 AM to 12 AM), odds ratio (** **95% CI** **)**							
	1 PM to 5 PM	1.17 (0.99-1.39)		1.18 (1.02-1.37)		1.12 (0.97-1.30)	
	6 PM to 8 PM	1.13 (0.84-1.51)		1.24 (0.96-1.6)		1.23 (0.96-1.57)	
	9 PM to 5 AM	0.44 (0.22-0.88)		0.45 (0.25-0.81)		0.43 (0.24-0.77)	
Observations, N			6224		5578		4945

^a^Only admissions between April 2019 and August 2019 and between April 2022 and August 2022 were considered.

^b^In addition to the variables listed here, the International Statistical Classification of Diseases, Tenth Revision chapters of the principal and secondary diagnoses were also included as control variables.

^c^Intervention period (T_1_): from April 1, 2022.

^d^DiD: difference-in-difference.

## Discussion

This study aims to explore the barriers and enablers of a digitalized discharge management system, to implement such a system using a logic model developed from these determinants, and to evaluate its impact on the readmission rate.

### Determinants and Implementation Strategies

The importance of the transmission of information for improved discharge management is also highlighted in the high rating of the strategies regarding the discharge letter, that is, developing an electronic discharge letter, continuously entering information into the letter, and always sending it to the GP. The discharge letter is the standard communication tool between inpatient and ambulatory care and found to be a source for deficits in information transfer [38]. In particular, delay and incompleteness of medication-related information endanger patients’ safety [39,40], leading to an increased risk of hospital readmission [41]. As shown for a sample of 20 Dutch hospitals, discharge letters vary in quality depending on patient and admission characteristics [42]. A standardized discharge letter can reduce transcription time and improve medical communication between physicians [43]. In addition, GPs prefer that discharge letters be written in a clear, concise, and understandable manner [44]. An electronic discharge letter generated from a computer-based document not only avoids transcription errors and lacks standardization but also ensures timely delivery [45]. In Germany, the discharge letter played a central role in approaches to creating a standard for intersectoral information exchange. For example, the VHitG (derived from the German “Verband der Hersteller von IT-Lösungen im Gesundheitswesen”) initiative “Intersectoral Communication” developed an implementation to facilitate the exchange of discharge letters between sectors, which is integrated into the existing IT system [46]. Another example is the recent approach by the National Association of Statutory Health Insurance Physicians and the German Hospital Association to create a standard for the electronic hospital discharge letter within the EHR [37].

To improve the standardization of the transmitted medication information, the use of the format of the nationwide medication plan was considered an important strategy in this study. In Germany, several projects have shown that physicians, pharmacists, and patients realize the benefits and accept the nationwide medication plan [47-49]. It can serve for the health care providers as a promising tool to improve the interdisciplinary and multiprofessional collaboration, especially as a digital solution that can realize its full potential [50]. Similar results have been reported in other countries [51,52]. In this study, participants suggested transferring medication-related information electronically and always in the format of the national medication plan. In the participating hospital, this strategy has been implemented during the project. For older patients in particular, shared medication records have the potential to reduce hospital readmissions [51].

Concerns about technical and temporal integrability were identified as an important barrier to the implementation of optimized discharge management. This includes an expected higher time consumption for the introduction of digitalized processes, a general fear of contact (owing to leaving known structures and a lack of electronic data processing experience), and further technical aspects (as a user-unfriendly system, electronic data processing errors, and interface problems). Similar barriers were identified in related eHealth projects [53-57]. Although the digitalization of processes was expected, in general, to be associated with time advantages, many of those involved associate the introduction with additional work effort. To overcome these concerns, successful implementation requires streamlining, simplifying, and redesigning the existing health care practices as a first step [58]. The strategy of introducing a physician-only hotline and a chat function for direct communication between the hospital and GPs could be seen as a simplification of communication instead of relying solely on the legally required discharge letter.

### Effect on Readmissions

A possible explanation for the low level of fidelity as well as the insignificant effect of SEKMA on readmissions could be the relatively short application period of half a year (from April 2022 to September 2022). Complex implementations such as those elaborated in SEKMA may require a longer time before they are applied in daily routines. Another reason for the insignificant effect on readmissions could be the rather good baseline level of the outcome in national comparison. Although other studies in Germany showed readmission rates, for example, of 18.1% (30 days) to 35.4% (90 days) for older patients (aged >65 years) [22], these rates were substantially lower for the patients in this study, that is, 11.8% (30 days) to 23.6% (90 days).

### Limitations

Our study had several limitations. First, the restrictions that existed owing to the COVID-19 pandemic might have affected the effectiveness of the implementation. All stakeholders involved in SEKMA faced a high workload owing to the pandemic as well as the requirements and measures resulting from the pandemic. However, the study results show that even under the special circumstances of the pandemic, it was possible to develop and implement an intersectoral optimization of discharge management. The infrastructure for the intersectoral care of patients created by the project has great potential to increase the quality of care, even if this could not yet be demonstrated with regard to readmissions. Future research should analyze the routine hospital data over the next 5 years.

Although the study included all relevant health care providers and considered the entire patient care pathway, the number of respondents from some professions may be rather small. For example, only 3 GPs were interviewed. However, the theoretical saturation effect in the statements made during the interviews suggests that this number is sufficient to identify the optimization potential as well as determinants.

### Conclusions

Creating a digital ecosystem that connects different health care providers seems to be a promising approach to ensure secure and fast networking of the sectors and to promote rapid information exchange between the sectors. The described intersectoral optimization of discharge management provides a structured template for the implementation of a similar local digital care networking infrastructure in other care regions in Germany and other countries with a similarly fragmented health care system.

## Appendix

Multimedia Appendix 1 COREQ (Consolidated Criteria for Reporting Qualitative Research) checklist.

Multimedia Appendix 2 Interview guide.

Multimedia Appendix 3 Supplemental tables with results of supplemental analyses.

## Abbreviations

COREQ: Consolidated Criteria for Reporting Qualitative Research
DiD: difference-in-difference
EHR: electronic health record
GP: general practitioner
ICD-10: International Statistical Classification of Diseases, Tenth Revision
KIM: Kommunikation im Medizinwesen
MIO: medical information object
SEKMA: Sektorübergreifende Optimierung des Entlassmanagements

## Data Availability

The data sets generated during and analyzed during this study are not publicly available due to the votum of the Ethics Committee of the University of Lübeck.
