# Supplementary material for: Designing and Implementation of a Digitalized Intersectoral Discharge Management System and Its Effect on Readmissions: Mixed Methods Approach
Source: J Med Internet Res. 2024 Mar 26;26:e47133. doi: 10.2196/47133 (PMC11005442; doi:10.2196/47133)
Supplement: Multimedia Appendix 2 [file jmir_v26i1e47133_app2.docx]

Interview guide

**Warmup**

1. Describe your role and related activities as well as processes in treating a patient in your department or facility. What do you know about the discharge management project SEKMA?

**Process analysis**

1. What information do you think is helpful to know about the patient to best treat or continue to care for the patient?
2. How are get information about the patient?
3. What is your experience with the transfer of information to you?
4. How do you communicate relevant information about the patient to aftercare or follow-up staff or facilities?
5. What areas could be improved in the transfer of information to follow-up care providers?

**Need for support**

1. How do you evaluate patient care in your department or facility? Do you see potential for optimizing the discharge management? How could this be optimized? What do you wish the hospital, nursing home, family members could do better? What do you think others would like you to do?

**Determinants**

An intersectoral discharge management system will be established in the hospital. The participating facilities should follow predefined and standardized processes and exchange their information about the patient with each other.

1. What do you expect from intersectoral discharge management, in which the participating institutions follow predefined and standardized processes and share their information about the patient with each other?
2. What has been your experience with sharing patient information across departments or facilities?
3. What do you think are the barriers to following such pre-defined processes and sharing patient information?
4. What do you think makes people more willing to adhere the predefined processes and share patient information?

**Closing**

1. What other aspect is important to you that we have not covered yet?
