# Supplementary material for: Designing and Implementation of a Digitalized Intersectoral Discharge Management System and Its Effect on Readmissions: Mixed Methods Approach
Source: J Med Internet Res. 2024 Mar 26;26:e47133. doi: 10.2196/47133 (PMC11005442; doi:10.2196/47133)
Supplement: Multimedia Appendix 3 [file jmir_v26i1e47133_app3.docx]

**Table S1: Characteristics of the interview participants**

|  | n (%) |
| --- | --- |
| **Total count** | 26 (100.0) |
| Hospital | 14 (53.8) |
| Medical doctors | 3 (11.5) |
| nurses | 4 (15.4) |
| Social workers | 4 (15.4) |
| Administration | 3 (11.5) |
| Nursing homes/ services | 9 (34.6) |
| GPs | 3 (11.5) |
| **Social demographics** |  |
| age (years), [IQR] | 42.4 [32-49] |
| female | 14 (53.8) |
| male | 12 (46.2) |
| **Ø interview duration [min,max]** | 33:11 min [20:19,59:36] |

IQR: interquartile range

**Table S2: Characteristics of the participants of the fidelity analysis**

| Facility | n (%) |
| --- | --- |
| hospital | 11(78.6) |
| outpatient nursing service | 1(7.1) |
| nursing home | 1(7.1) |
| medical practice | 1(7.1) |
| Activity |  |
| in the social service | 0(0.0) |
| as a medical assistant | 0(0.0) |
| in a nursing profession | 2(14.3) |
| in a medical profession | 12(85.7) |
| Satisfaction with the change in discharge management? |  |
| very satisfied | 1(7.7) |
| satisfied | 3(23.1) |
| neither | 4(30.8) |
| dissatisfied | 4(30.8) |
| very dissatisfied | 1(7.7) |

**Table S3: Application in routine, n/N(%)**

| **No.** | **Strategy** | **yes**  **(always)** | **partly** | **no (never)** |
| --- | --- | --- | --- | --- |
| 1 | Always send discharge letter to the GP | 7/11(63.6) | 2/11(18.2) | 2/11(18.2) |
| 2 | Providing mobile devices to the hospital’s social services department (e.g., laptop, tablet) | 7/8(87.5) | 1/8(12.5) | 0/8(0) |
| 3 | Generating individual medication plans in the format of the national medication plan | 11/13(84.6) | 1/13(7.7) | 1/13(7.7) |
| 6 | Standardized consent process for discharge management already upon admission | 5/7(71.4) | 1/7(14.3) | 1/7(14.3) |
| 7 | Sending/receiving an electronic discharge letter (e.g., compatible with the electronic patient record) | 2/12(16.7) | 1/12(8.3) | 9/12(75.0) |
| 15 | Hotline used exclusively by physicians for direct exchange between hospital and GPs | 2/10(20.0) | 4/10(40.0) | 4/10(40.0) |
| 17 | Continuous digital maintenance of a potential discharge letter (e.g., in preparation for an unplanned discharge) | 4/12(33.3) | 4/12(33.3) | 4/12(33.3) |

**Table S4: (Re)admissions by department**

|  | | **total hospital** | *Intervention group* | *Control group* | |
| --- | --- | --- | --- | --- | --- |
|  | |  | **vascular surgery** | **cardiology** | **medical clinic** |
| **Admissions, n(%**^a^**)** | | |  |  |  |
|  | patients | 12407 | 637 (5.1) | 1680 (13.5) | 3726 (30.0) |
|  | cases | 14854 | 759 (5.1) | 1895 (12.8) | 4175 (28.1) |
| **Readmission after days, n/N(%**^b^**)** | | | |  |  |
| 30 | patients | 994/11386 (8.7) | 76/602 (12.6) | 160/1528 (10.5) | 369/3456 (10.7) |
|  | cases | 1222/13477 (9.1) | 80/705 (11.3) | 176/1700 (10.4) | 423/3850 (11.0) |
| 60 | patients | 1406/10217 (13.8) | 132/550 (24.0) | 240/1372 (17.5) | 489/3103 (15.8) |
|  | cases | 1799/11975 (15.0) | 148/637 (23.2) | 269/1516 (17.7) | 582/3451 (16.9) |
| 90 | patients | 1542/9016 (17.1) | 142/487 (29.2) | 270/1227 (22.0) | 564/2746 (20.5) |
|  | cases | 1975/10478 (18.8) | 161/560 (28.8) | 303/1344 (22.5) | 661/3048 (21.7) |

Only admissions between April 2019 to August 2019 and April 2022 to August 2022 were considered. ^a^Percentage of patients/cases admitted to the department of all patients/cases admitted to the hospital. ^b^Readmission rate

**Table S5: Risk factors for re-admissions**

|  | **all**  **cases** | **Readmissions after** | | | | | |
| --- | --- | --- | --- | --- | --- | --- | --- |
|  |  | **30 days** | | **60 days** | | **90 days** | |
|  | n  (%) | n  (%) | OR  (P-values) | n  (%) | OR  (P-values) | n  (%) | OR  (P-values) |
| **social demographics** |  |  |  |  |  |  |  |
| female | 3062 (48.8) | 323 (10.5) | 0.94  (.45) | 473 (17.2) | 0.91  (.20) | 531 (21.9) | 0.91 (.17) |
| male | 3193 (51.2) | 356 (11.1) | 1.06  (.45) | 526 (18.5) | 1.09  (.20) | 594 (23.5) | 1.1  (.17) |
| aged below 65 | 1715 (27.9) | 126 (7.3) | 0.57 (<.001) | 197 (12.8) | 0.6 (<.001) | 218 (16.1) | 0.57 (<.001) |
| aged 65+ | 4440 (70.6) | 537 (12.1) | 1.62 (<.001) | 780 (19.6) | 1.57 (<.001) | 881 (25.1) | 1.64 (<.001) |
| **length of stay** |  |  |  |  |  |  |  |
| <3 days | 1538 (25.3) | 96 (6.2) | 0.47 (<.001) | 150 (10.9) | 0.49 (<.001) | 171 (14.3) | 0.49 (<.001) |
| 3-5 days | 1973 (31.4) | 188 (9.5) | 0.81  (.02) | 282 (15.7) | 0.81 (.005) | 325 (20.4) | 0.82 (0.01) |
| 6-9 days | 1510 (24.2) | 199 (13.2) | 1.35 (<.001) | 296 (22.1) | 1.44 (<.001) | 323 (27.2) | 1.38 (<.001) |
| >10 days | 1234 (19.0) | 196 (15.9) | 1.77 (<.001) | 271 (24.6) | 1.69 (<.001) | 306 (31.2) | 1.74 (<.001) |
| **transfer within the hospital** |  |  |  |  |  |  |  |
| Transfer between departments | 1354 (21.2) | 176 (13) | 1.31  (.004) | 251 (20.6) | 1.26  (.004) | 286 (26.4) | 1.29 (.001) |
| Intensive care | 264 (4.2) | 19 (7.2) | 0.63  (.05) | 35 (15.8) | 0.86  (.41) | 44 (22.2) | 0.97  (.87) |
| **Discharge time** |  |  |  |  |  |  |  |
| 6am-12 am | 2852 (45.3) | 302 (10.6) | 0.95  (.54) | 450 (17.4) | 0.95  (.49) | 521 (22.6) | 0.99  (.89) |
| 1pm - 5pm | 2647 (42.5) | 305 (11.5) | 1.13  (.15) | 442 (18.7) | 1.11  (.14) | 485 (23.3) | 1.06 (.38) |
| 6pm - 8pm | 585 (9.4) | 63 (10.8) | 0.99  (.94) | 94 (18.5) | 1.05  (.69) | 105 (23.5) | 1.05 (.66) |
| 9pm - 5am | 171 (2.7) | 9  (5.3) | 0.45  (.02) | 13  (8.5) | 0.42  (.003) | 14 (11.2) | 0.42 (.003) |

Only admissions between April 2019 to August 2019 and April 2022 to August 2022 were considered. OR: Odds Ratio estimated by bivariate logistic regression models

**Table S5: Risk factors for re-hospitalization (continued)**

|  |  | **total**  **cases** | **Readmissions after** | | | | | |
| --- | --- | --- | --- | --- | --- | --- | --- | --- |
|  |  |  | **30 days** | | **60 days** | | **90 days** | |
| **ICD-10** | **Description** | **n**  **(%)** | **n**  **(%)** | **OR**  **(P-values)** | **n**  **(%)** | **OR**  **(P-values)** | **n**  **(%)** | **OR**  **(P-values)** |
| A00-B99 | Certain infectious and parasitic diseases | 396 (6.2) | 36 (9.1) | 0.81  (.24) | 50 (14.4) | 0.76  (.08) | 54 (17.7) | 0.72  (.03) |
| C00-D48 | Neoplasms | 421 (6.3) | 98 (23.3) | 2.74  (<.001) | 121 (31.7) | 2.29 (<.001) | 130 (38.1) | 2.24 (<.001) |
| D50-D90 | Diseases of the blood and blood-forming organs and certain disorders involving the immune mechanism | 56  (1.0) | 4  (7.1) | 0.63  (.37) | 7 (13.7) | 0.73  (.44) | 8 (17.4) | 0.71  (.39) |
| E00-E90 | Endocrine, nutritional and metabolic diseases | 262 (4.2) | 31 (11.8) | 1.11  (.60) | 52 (22.8) | 1.38  (.046) | 55 (28.2) | 1.35  (.06) |
| F00-F99 | Mental and behavioural disorders | 148 (2.2) | 13 (8.8) | 0.79  (.41) | 21 (15.3) | 0.83  (.44) | 22 (17.6) | 0.72  (.17) |
| G00-G99 | Diseases of the nervous system | 147 (2.3) | 12 (8.2) | 0.73  (.29) | 15 (11.6) | 0.6  (.07) | 18 (16.4) | 0.66  (.11) |
| H00-H59 | Diseases of the eye and adnexa | 3  (0.0) | 0 | - | 0 | - | 0 | - |
| H60-H95 | Diseases of the ear and mastoid process | 21  (0.3) | 0 | - | 0 | - | 1  (6.3) | 0.23  (.15) |
| I00-I99 | Diseases of the circulatory system | 2619 (42.2) | 262 (10.0) | 0.86  (.07) | 420 (17.9) | 1.01  (.92) | 478 (22.8) | 1  (.96) |
| J00-J99 | Diseases of the respiratory system | 572  (9.0) | 60 (10.5) | 0.96  (.77) | 80  (16.0) | 0.87  (.27) | 91 (20.5) | 0.87  (.25) |
| K00-K93 | Diseases of the digestive system | 793 (12.9) | 83 (10.5) | 0.95  (.71) | 129 (17.7) | 0.99  (.91) | 146 (23.0) | 1.02  (.86) |
| L00-L99 | Diseases of the skin and subcutaneous tissue | 29  (0.5) | 5 (17.2) | 1.72  (.27) | 7  (25.0) | 1.54  (.32) | 8 (28.6) | 1.36  (.46) |
| M00-M99 | Diseases of the musculoskeletal system and connective tissue | 82  (1.2) | 9 (11.0) | 1.01  (.97) | 11 (13.8) | 0.73  (.34) | 9 (12.7) | 0.49  (.046) |
| N00-N99 | Diseases of the genitourinary system | 230 (3.7) | 31 (13.5) | 1.29  (.19) | 40 (20.1) | 1.17  (.39) | 50 (29.1) | 1.41  (.04) |
| O00-O99 | Pregnancy, childbirth and the puerperium | 5  (0.1) | 0 | - | 0 | - | 0 | - |
| P00-P96 | Certain conditions originating in the perinatal period | 0 | 0 | - | 0 | - | 0 | - |
| Q00-Q99 | Congenital malformations, deformations and chromosomal abnormalities | 2  (0.0) | 0 | - | 0 | - | 0 | - |
| R00-R99 | Symptoms, signs and abnormal clinical and laboratory findings, not elsewhere classified | 247 (4.2) | 17 (6.9) | 0.6  (.04) | 20 (9.3) | 0.46  (.001) | 26 (14.4) | 0.56  (.01) |
| S00-T98 | Injury, poisoning and certain other consequences of external causes | 179  (3.0) | 16 (8.9) | 0.8  (.40) | 24 (14.5) | 0.77  (.25) | 26 (18.1) | 0.74  (.18) |
| V01-Y98 | External causes of morbidity and mortality | 0 | 0 | - | 0 | - | 0 | - |
| Z00-Z99 | Factors influencing health status and contact with health services | 43  (0.6) | 2  (4.7) | 0.4  (.21) | 2  (5.3) | 0.25  (.06) | 3  (9.1) | 0.34  (.07) |
| U00-U99 | Codes for special purposes | 0 | 0 | - | 0 | - | 0 | - |

Only admissions between April 2019 to August 2019 and April 2022 to August 2022 were considered. OR: Odds Ratio estimated by bivariate logistic regression models

**Table S6: Bivariate analysis of the intervention effect for patients aged 65+**

| **Baseline (**$\boldsymbol{T}_{\boldsymbol{0}}$**): 01.04.19 – 31.08.19**  **Intervention (**$\boldsymbol{T}_{\boldsymbol{1}}$**): from 01.04.2022** | **Intervention group:**  vascular surgery | **Control group:**  internal medicine, cardiology |
| --- | --- | --- |
| **Re-Adm. 30 days:** $\boldsymbol{T}_{\boldsymbol{1}}$ **until 03.08.22** | n=504 | n=3936 |
| Baseline ($T_{0}$), n /N (%) | 36/306(11.8) | 281/2261(12.4) |
| Intervention ($T_{1}$), n /N (%) | 29/198(14.6) | 191/1675(11.4) |
| Difference $T_{0}$ & $T_{1}$(*P-*value) | 2.88(.36) | -1.03(.33) |
| **DiD: Δ (*P-*value)** | **3.91(.24)** | |
| **Re-Adm. 60 days:** $\boldsymbol{T}_{\boldsymbol{1}}$ **until 04.07.22** | n=452 | n=3523 |
| Baseline ($T_{0}$), n /N (%) | 77/306(25.2) | 430/2261(19.0) |
| Intervention ($T_{1}$), n /N (%) | 37/146(25.3) | 236/1262(18.7) |
| Difference $T_{0}$ & $T_{1}$(P-value) | 0.18(.97) | -0.32(.82) |
| **DiD: Δ (P-value)** | **0.5(.91)** | |
| **Re-Adm. 90 days:** $\boldsymbol{T}_{\boldsymbol{1}}$ **until 04.06.22** | n=397 | n=3116 |
| Baseline ($T_{0}$), n /N (%) | 96/306(31.4) | 552/2261(24.4) |
| Intervention ($T_{1}$), n /N (%) | 30/91(33.0) | 203/855(23.7) |
| Difference $T_{0}$ & $T_{1}$(P-value) | 1.59(.78) | -0.67(.70) |
| **DiD: Δ (P-value)** | **2.27(.70)** | |

Only admissions between April 2019 to August 2019 and April 2022 to August 2022 were considered. DiD: Difference-in-Difference, $\Delta=\left( y_{{IT}_{0}}- y_{{IT}_{1}} \right)-(y_{CT_{0}}- y_{{CT}_{1}})$, where $y_{{\boldsymbol{\cdot}T}_{0}}$and $y_{{\boldsymbol{\cdot}T}_{1}}$are the readmission rate of the intervention ($I$) and the control group ($C$) at $T_{0}$ and $T_{1}$, respectively.

**Table S7: Multivariate Logistic Regression for patients aged 65+, Odds Ratios (*P*-values)**

|  | **Readmissions after** | | |
| --- | --- | --- | --- |
| **Variable** | **30 days**  $\boldsymbol{T}_{\boldsymbol{1}}$ **until 03.08.22** | **60 days**  $\boldsymbol{T}_{\boldsymbol{1}}$ **until 04.07.22** | **90 days**  $\boldsymbol{T}_{\boldsymbol{1}}$ **until 04.06.22** |
| **SEKMA (DiD), [95% confidence interval]** | **1.54**  **(0.14) [0.87;2.72]** | **1.08**  **(0.77)**  **[0.66;1.76]** | **1.15**  **(0.61)**  **[0.67;1.97]** |
| post-intervention | 0.93  (.47) | 1.01  (.91) | 0.99  (.89) |
| intervention group | 0.94  (.76) | 1.36  (.05) | 1.33  (.05) |
| **social demographics** |  |  |  |
| female | 0.96  (.68) | 0.93  (.36) | 0.89  (.15) |
| aged 65+ | - | - | - |
| **length of stay** (reference: <3 days) |  |  |  |
| 3-5 days | 1.39  (.04) | 1.25  (.09) | 1.21  (.12) |
| 6-9 days | 1.96 (<.001) | 1.75  (<.001) | 1.58  (<.001) |
| >10 days | 2.30  (<.001) | 1.88  (<.001) | 1.81  (<.001) |
| **transfer within the hospital** |  |  |  |
| Transfer between departments | 0.94  (.57) | 0.97  (.79) | 0.99  (.91) |
| Intensive care | 0.34  (.001) | 0.64  (.06) | 0.66  (.06) |
| **Discharge time** (reference: 6am-12 am) |  |  |  |
| 1pm - 5pm | 1.23  (.04) | 1.21  (.03) | 1.12  (.17) |
| 6pm - 8pm | 1.23  (.21) | 1.23  (.17) | 1.20  (.22) |
| 9pm - 5am | 0.46  (.04) | 0.39  (.005) | 0.34  (.002) |
| Observations | 4428 | 3965 | 3513 |

Only admissions between April 2019 to August 2019 and April 2022 to August 2022 were considered. DiD: Difference-in-Difference. In addition to the variables listed here, the ICD chapters of the principal and secondary diagnoses were also included as control variables.

**Table S8: Multivariate Logistic Regression** **of vascular surgery, Odds Ratios (*P*-values)**

|  |  | **Readmissions after** | | |
| --- | --- | --- | --- | --- |
| **Variable** |  | **30 days**  $\boldsymbol{T}_{\boldsymbol{1}}$ **until 03.08.22** | **60 days**  $\boldsymbol{T}_{\boldsymbol{1}}$ **until 04.07.22** | **90 days**  $\boldsymbol{T}_{\boldsymbol{1}}$ **until 04.06.22** |
| SEKMA (Post-Intervention), [95% confidence interval] |  | 1.37  (.21) [0.83;2.25] | 1.23  (.33)  [0.82;1.85] | 1.37  (.17)  [0.87;2.16] |
| **social demographics** |  |  |  |  |
| female |  | 0.95  (.85) | 1.02  (.93) | 0.99  (.96) |
| aged 65+ |  | 1.60  (.13) | 1.39  (.15) | 1.60  (.04) |
| **length of stay** (reference: <3 days) |  |  |  |  |
| 3-5 days |  | 1.82  (.18) | 1.36  (.35) | 1.00  (.99) |
| 6-9 days |  | 1.25  (.64) | 1.40  (.31) | 1.13  (.71) |
| >10 days |  | 2.33  (.05) | 1.91  (.04) | 1.52  (.17) |
| **transfer within the hospital** |  |  |  |  |
| Transfer between departments |  | 1.18  (.72) | 1.09  (.83) | 1.27  (.55) |
| Intensive care |  | 0.54  (.26) | 0.60  (.22) | 0.71  (.38) |
| **Discharge time** (Reference: 6am-12 am) |  |  |  |  |
| 1pm - 5pm |  | 1.72  (.04) | 1.43  (.08) | 1.80  (.004) |
| 6pm - 8pm |  | 1.50  (.48) | 2.95  (.01) | 2.92  (.03) |
| 9pm - 5am |  | 0.82  (.85) | 0.37  (.36) | 0.34  (.33) |
| Observations |  | 680 | 627 | 550 |

Based on observations of vascular surgery only. Only admissions between April 2019 to August 2019 and April 2022 to August 2022 were considered. DiD: Difference-in-Difference. In addition to the variables listed here, the ICD chapters of the principal and secondary diagnoses were also included as control variables.
